# Supplementary material for: All-cause and cause-specific mortality during and following incarceration in Brazil: A retrospective cohort study
Source: PLoS Med. 2021 Sep 17;18(9):e1003789. doi: 10.1371/journal.pmed.1003789 (PMC8486113; doi:10.1371/journal.pmed.1003789)
Supplement: S1 Table — List of analyses and figures, organized by incarceration status (during incarceration or following release), group (men, boys, or women), and facility type or location. Reference population used for computing IRRs is shown. IRR, incidence rate ratio. (PDF) [file pmed.1003789.s003.pdf]

<table>
<tr><th colspan="2">Population</th><th>Facility type/location</th><th>Analyses (figure)</th><th>Reference population</th></tr>
<tr><td rowspan="5">Incarcerated</td><td rowspan="3">Men</td><td>Closed prisons</td><td>Age-specific (Fig. S6) and age-standardized (Fig. 3) rates; instantaneous hazard (Fig. S8) and binned age-standardized rates (Fig. S9)</td><td rowspan="3">Non-incarcerated men</td></tr>
<tr><td>Semi-open prisons</td><td>Age-specific (Fig. S6) and age-standardized (Fig. S7) rates; instantaneous hazard (Fig. S8) and binned age-standardized rates (Fig. S9)</td></tr>
<tr><td>Police stations</td><td>Age-specific (Fig. S6) and age-standardized (Fig. S7) rates; instantaneous hazard (Fig. S8) and binned age-standardized rates (Fig. S9)</td></tr>
<tr><td>Boys</td><td>Youth detention</td><td>Crude (age-specific) rates (Fig. S7); instantaneous hazard (Fig. S8) and binned rates (Fig. S9)</td><td>Non-incarcerated boys (age 14-19)</td></tr>
<tr><td>Women</td><td>All facility types (closed prisons, semi-open prisons, police stations)</td><td>Age-standardized rates (Fig. 3)</td><td>Non-incarcerated women</td></tr>
<tr><td rowspan="2">Post-release</td><td>Men</td><td>Released from prison (closed or semi-open)</td><td>Age-specific (Fig. S6) and age-standardized (Fig. 4) rates; instantaneous hazard (Fig. 5); Kaplan-Meier survival, cause-of-death, and time-to-death, stratified by total time incarcerated (Fig. S11)</td><td>Non-incarcerated men</td></tr>
<tr><td>Women</td><td>Released from prison (closed or semi-open)</td><td>Age-specific (Fig. S10A) and age-standardized (Fig. 4) rates; instantaneous hazard (Fig. S10B)</td><td>Non-incarcerated women</td></tr>
</table>
