# Supplementary material for: All-cause and cause-specific mortality during and following incarceration in Brazil: A retrospective cohort study
Source: PLoS Med. 2021 Sep 17;18(9):e1003789. doi: 10.1371/journal.pmed.1003789 (PMC8486113; doi:10.1371/journal.pmed.1003789)
Supplement: S2 Table — Causes are sorted top to bottom by total number of deaths among all groups. Proportions of deaths resulting from each cause within each group (columns) are indicated in parentheses. Top 5 causes of death within each group are indicated by a number sign (#). Percentages may not sum to 100% due to rounding. TB, tuberculosis. (PDF) [file pmed.1003789.s004.pdf]

| | Incarcerated Men and Boys | Formerly Incarcerated Men and Boys | Incarcerated Women | Formerly Incarcerated Women | All groups |
|---|---|---|---|---|---|
| **All causes** | 672 | 2236 | 33 | 186 | 3127 |
| **Interpersonal violence** | 226 (33.6%)# | 848 (37.9%)# | 2 (6.1%)# | 31 (16.7%)# | 1107 (35.4%)# |
| **Cardiovascular disease** | 79 (11.8%)# | 311 (13.9%)# | 7 (21.2%)# | 29 (15.6%)# | 426 (13.6%)# |
| **Transport injuries** | 28 (4.2%) | 205 (9.2%)# | 0 | 7 (3.8%) | 240 (7.7%)# |
| **Neoplasms** | 24 (3.6%) | 139 (6.2%)# | 3 (9.1%)# | 34 (18.3%)# | 200 (6.4%)# |
| **Suicide** | 55 (8.2%)# | 90 (4.0%)# | 3 (9.1%)# | 7 (3.8%) | 155 (5.0%)# |
| **Respiratory infections (including TB)** | 50 (7.4%)# | 78 (3.5%) | 0 | 8 (4.3%)# | 136 (4.3%) |
| **HIV/AIDS** | 30 (4.5%)# | 79 (3.5%) | 7 (21.2%)# | 11 (5.9%)# | 127 (4.1%) |
| **Other/unknown causes** | 180 (26.8%) | 486 (21.7%) | 11 (33.3%) | 59 (31.7%) | 736 (23.5%) |
