# Supplementary figures and images for: All-cause and cause-specific mortality during and following incarceration in Brazil: A retrospective cohort study
Source: PLoS Med. 2021 Sep 17;18(9):e1003789. doi: 10.1371/journal.pmed.1003789 (PMC8486113; doi:10.1371/journal.pmed.1003789)

## Same RGI number; multiple names

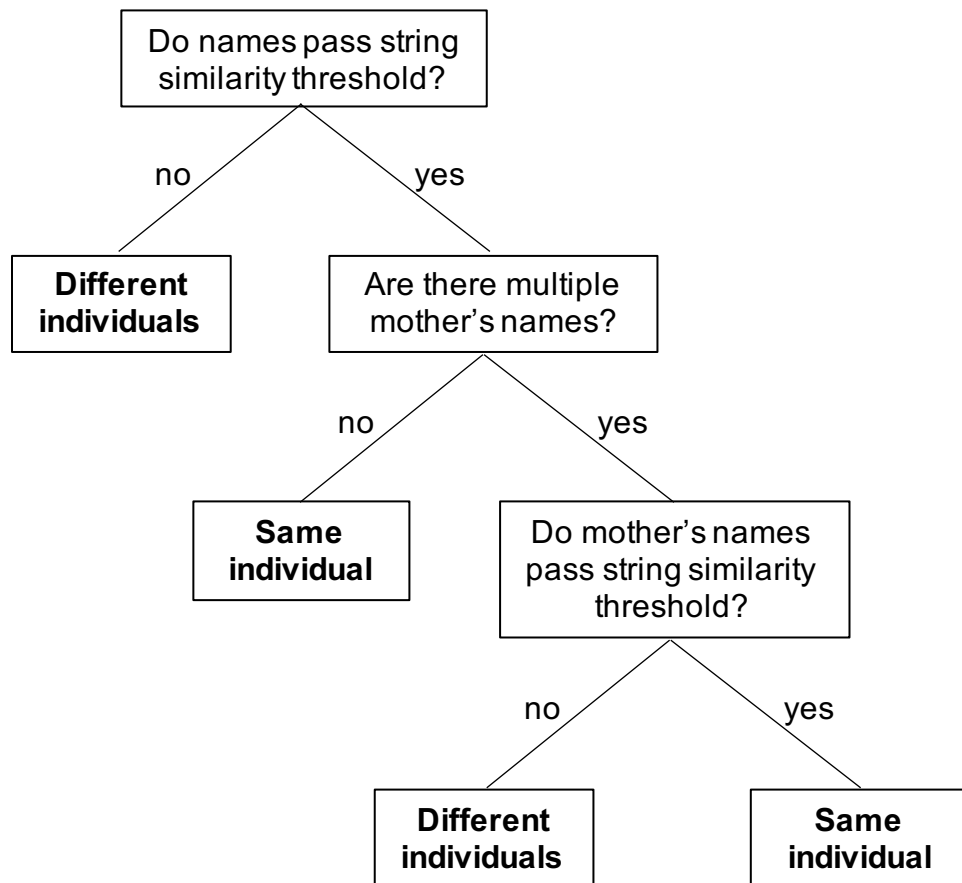

## Same name, multiple RGI numbers

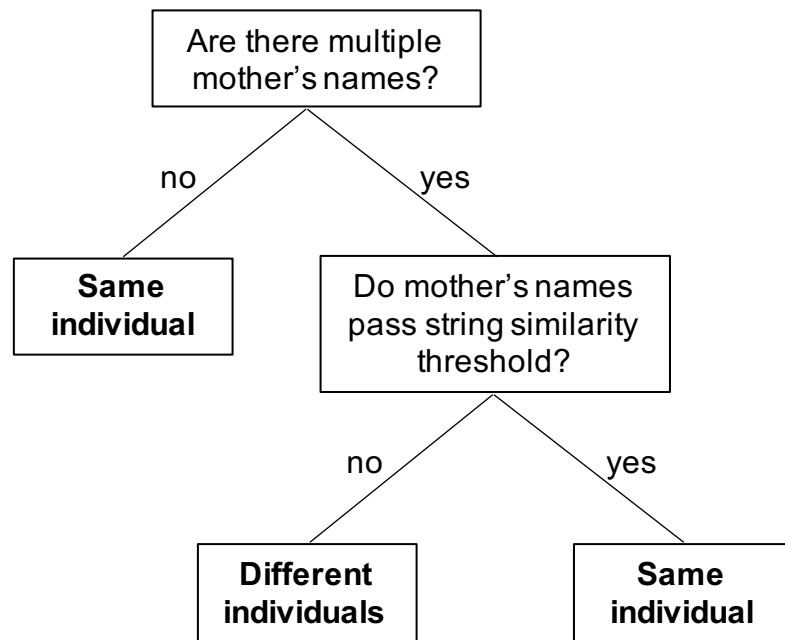

Supplement: S1 Fig — Logic for determining whether a set of SIGO records correspond to the same unique individual in the case of records with (A) the same RGI number (RGI, an internal identification number) but multiple names or (B) the same name but multiple RGI numbers. String similarity between names and mother’s names was computed using Levenshtein distance, weighted based on difference in length between strings. RGI, Registro Geral do Interno; SIGO, Sistema Integrado de Gestão Operacional. (PDF) [file pmed.1003789.s005.pdf]

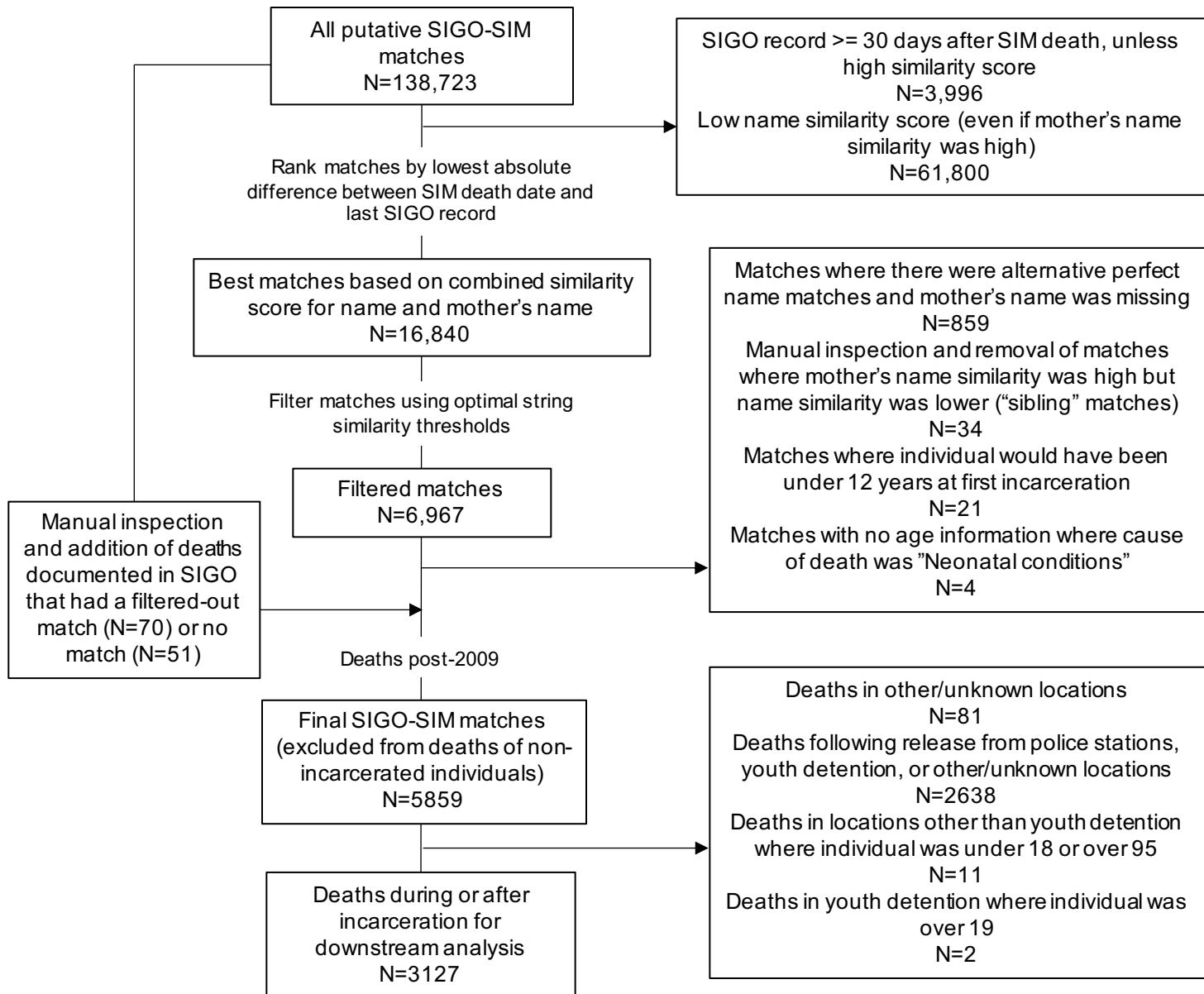

Supplement: S2 Fig — Arrows to the far right indicate exclusion. SIGO, Sistema Integrado de Gestão Operacional; SIM, Sistema de Informações Sobre Mortalidade. (PDF) [file pmed.1003789.s006.pdf]

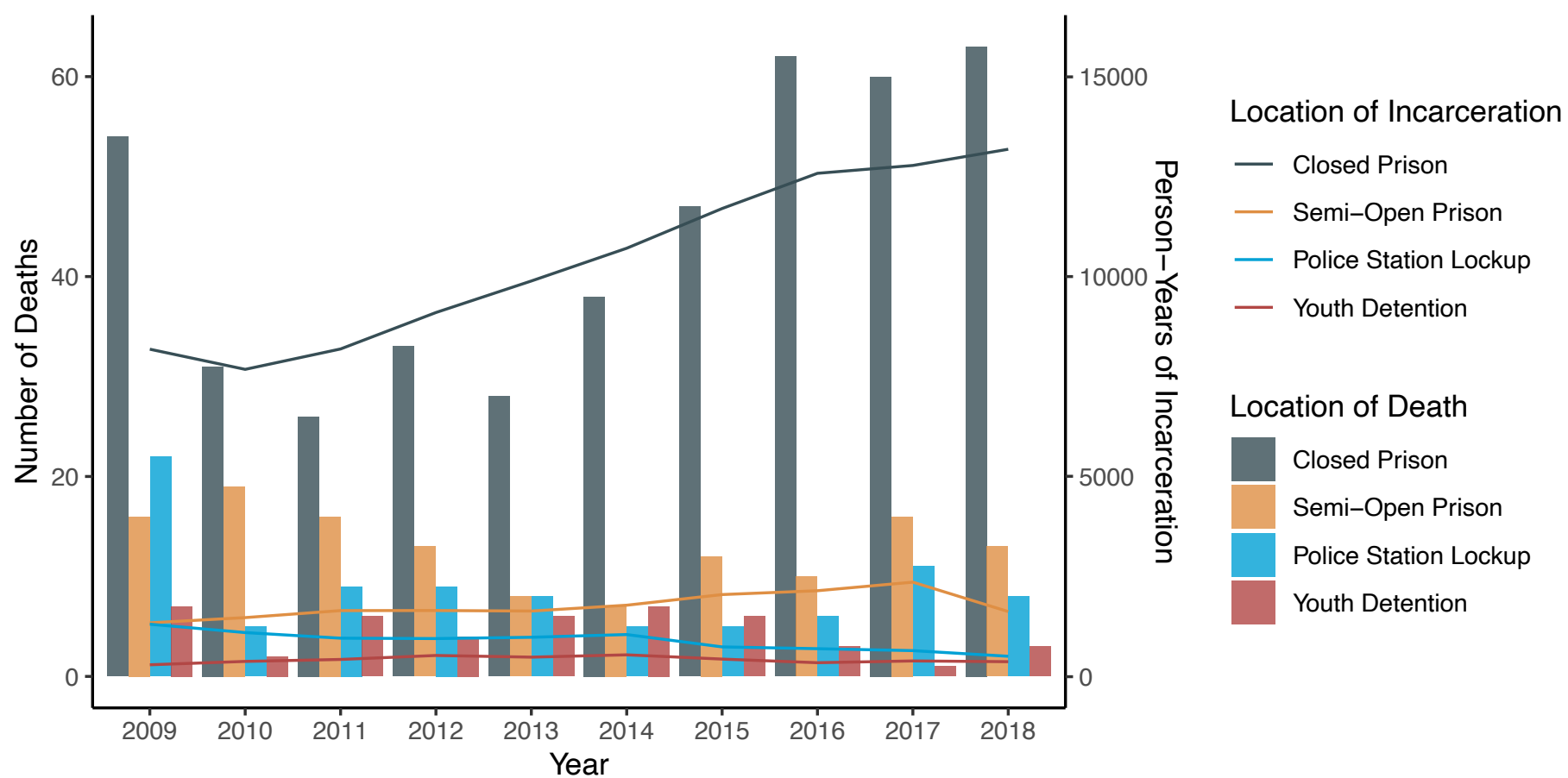

Supplement: S3 Fig — Bar plots depicting number of deaths of individuals during incarceration in each facility type (left axis), overlaid with line plots of person-years of incarceration in each facility type (right axis). (PDF) [file pmed.1003789.s007.pdf]

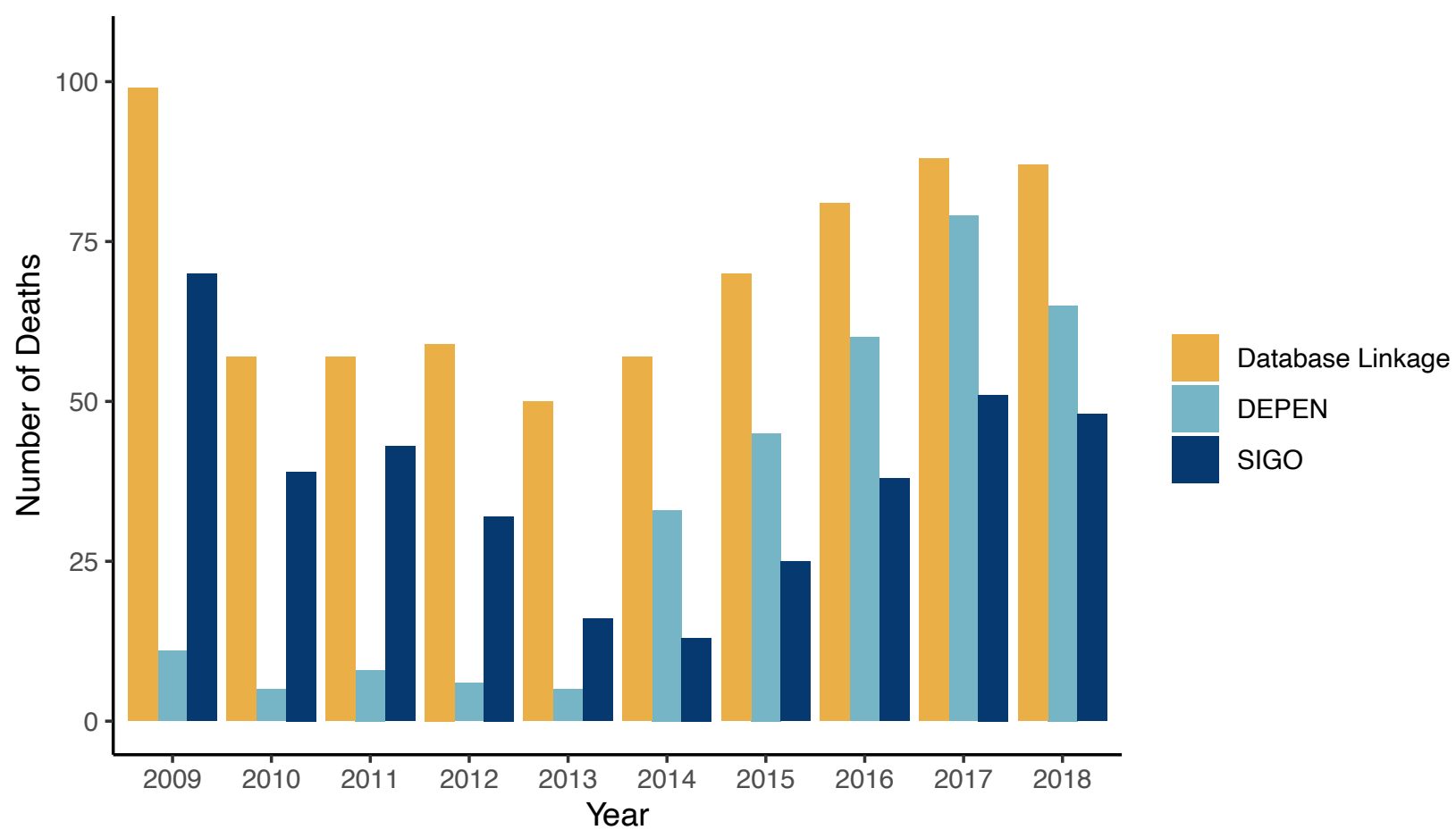

Supplement: S4 Fig — Bar plot of deaths during incarceration each year identified through database linkage in this study (yellow), reported by the Brazilian National Prison Department (DEPEN; light blue), or documented in Mato Grosso Do Sul’s incarceration database (SIGO; dark blue). DEPEN, Departamento Penitenciário Nacional; SIGO, Sistema Integrado de Gestão Operacional. (PDF) [file pmed.1003789.s008.pdf]

## Closed Prison

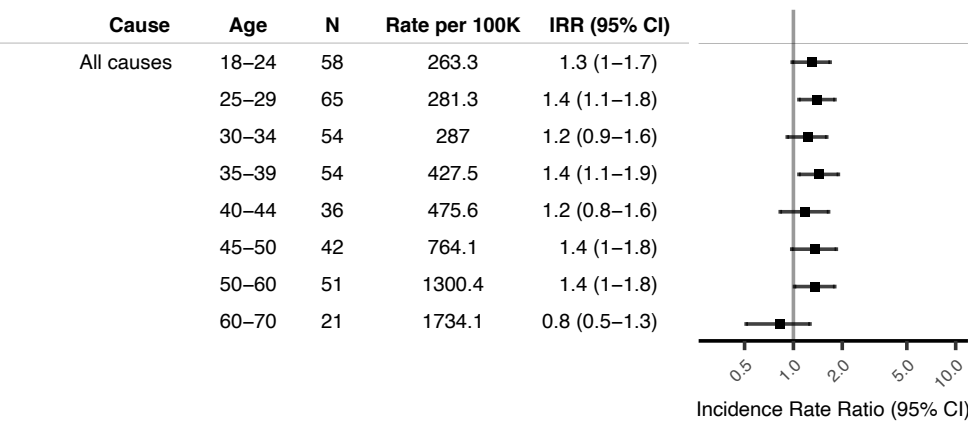

## Semi-Open Prison

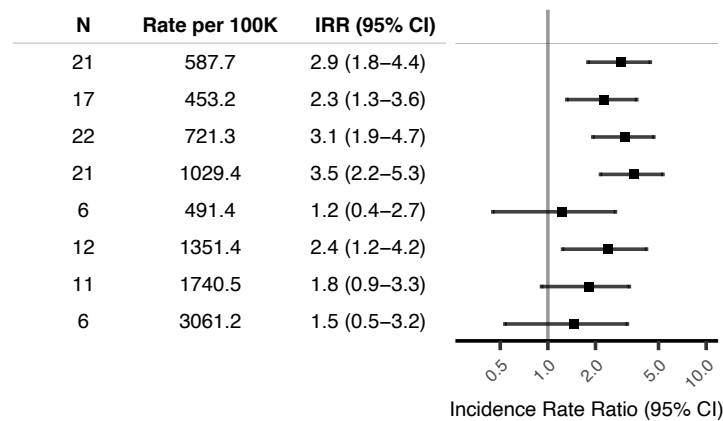

## Police Station

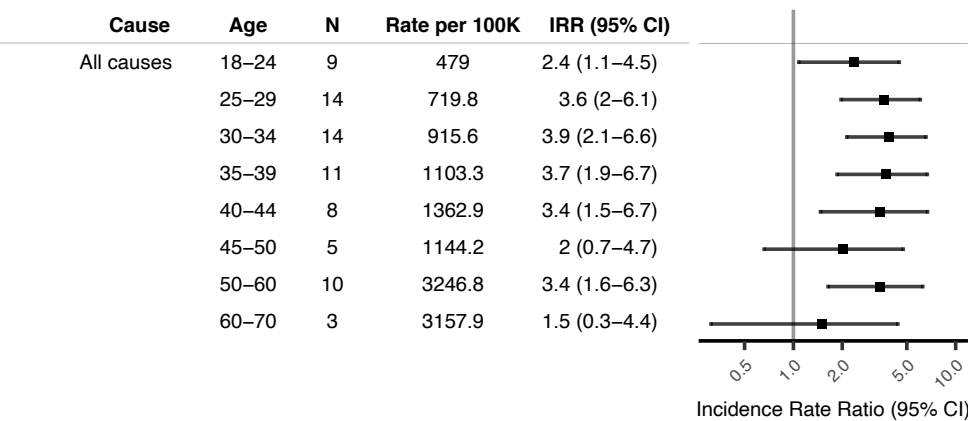

## Post-Release

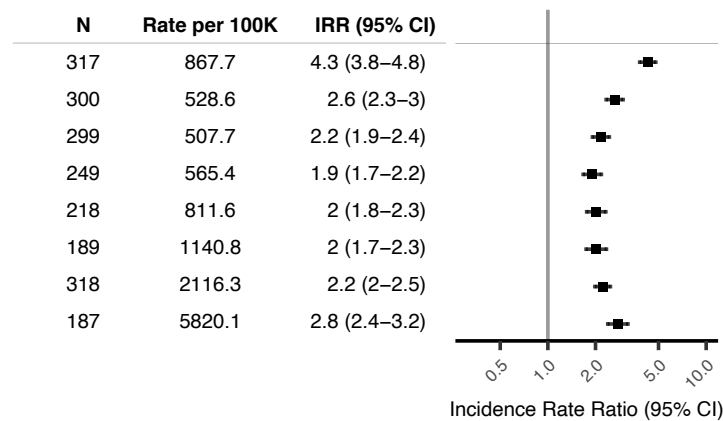

Supplement: S6 Fig — Age-specific all-cause mortality rates per 100,000 person-years and IRRs for incarcerated and formerly incarcerated men. IRRs were computed relative to non-incarcerated male Mato Grosso do Sul residents. 100K, 100,000 person-years; IRR, incidence rate ratio; N, number of deaths. (PDF) [file pmed.1003789.s010.pdf]

**A**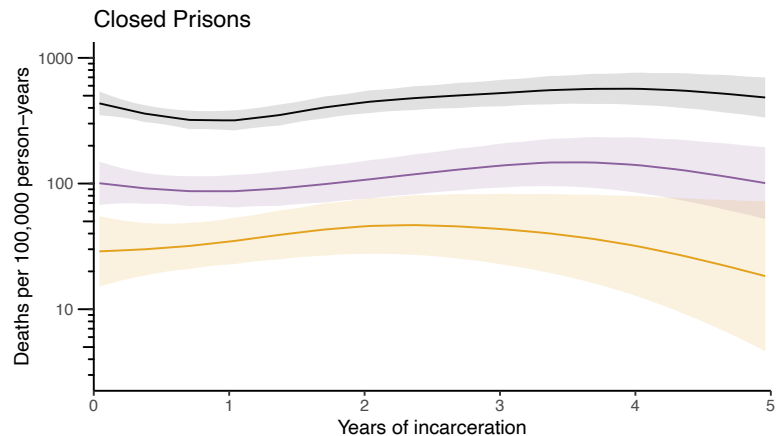**B**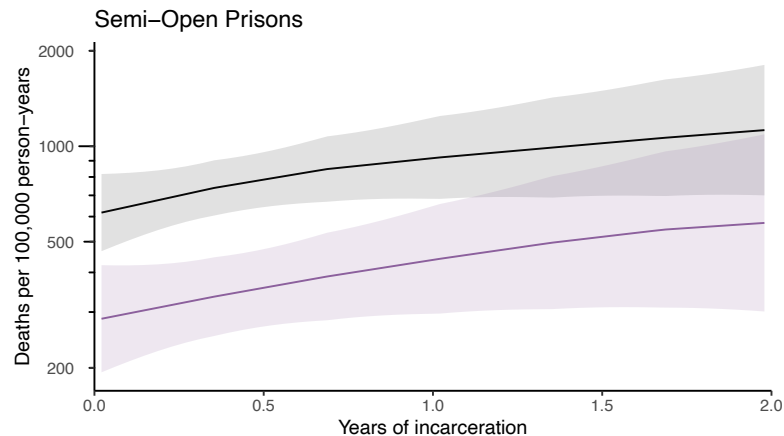**C**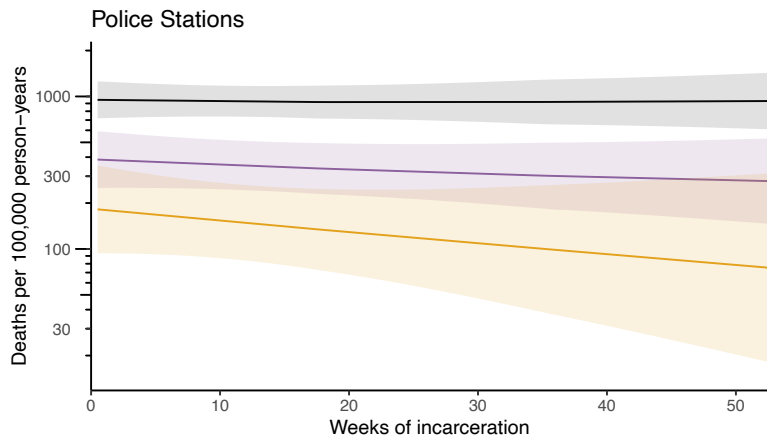**D**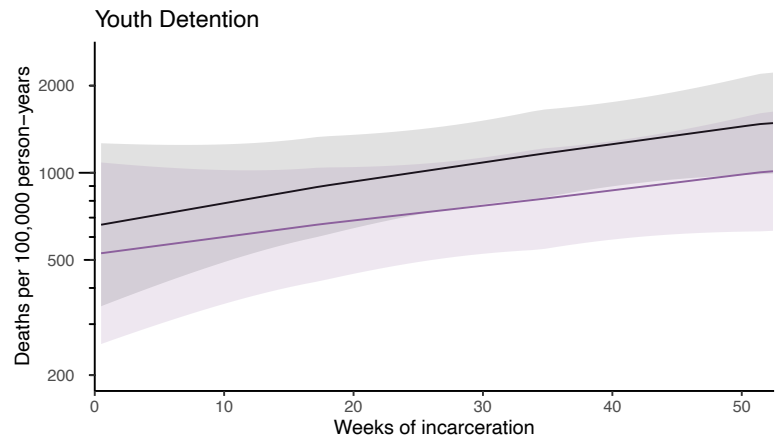

— All causes — Violence — Suicide

Supplement: S8 Fig — Instantaneous death rate among incarcerated men and boys in for all causes of death (black), violent deaths (purple), or suicide (yellow) in (A) closed prisons, (B) semi-open prisons, (C) police stations, and (D) youth detention. Bands indicate the 95% CI. Y-axis is shown in log10 scale and does not start at 1. Suicide rate not shown for semi-open prisons or youth detention due to the low number of suicides in these facilities. CI, confidence interval. (PDF) [file pmed.1003789.s012.pdf]

**A****Closed Prison**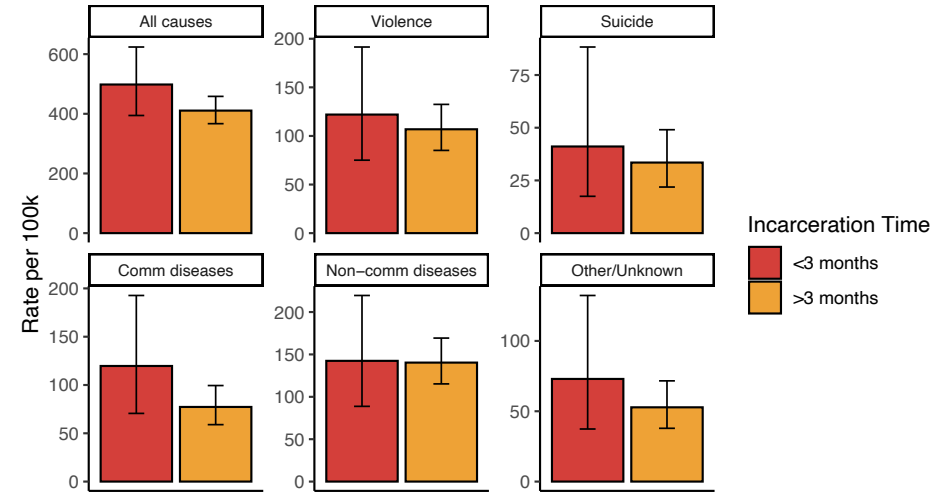**B****Semi-Open Prison**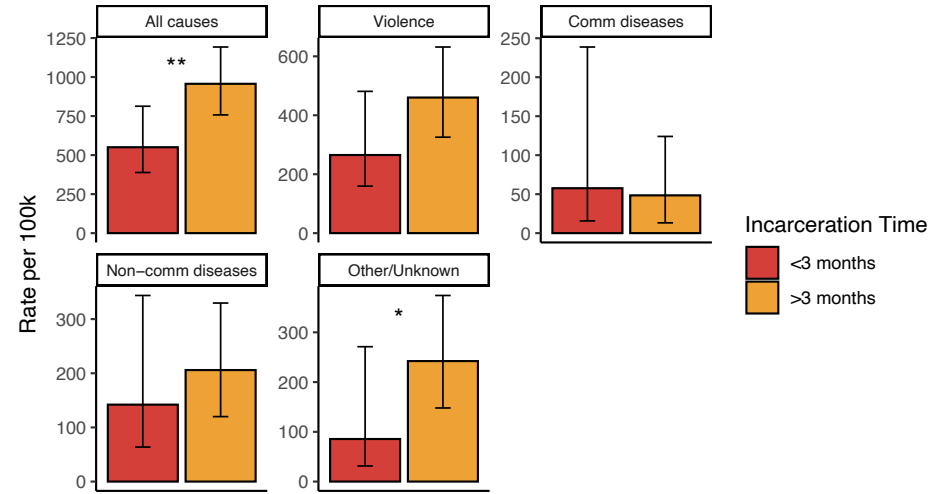**C****Police Station**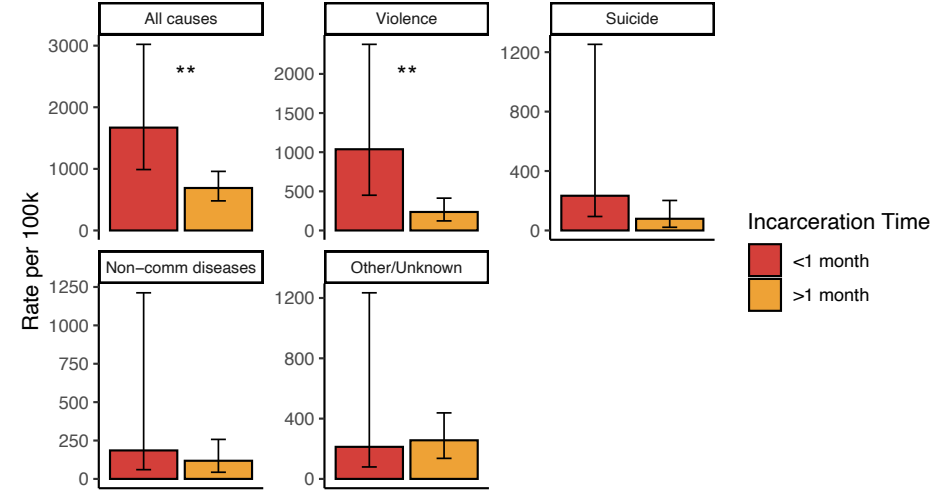**D****Youth Detention**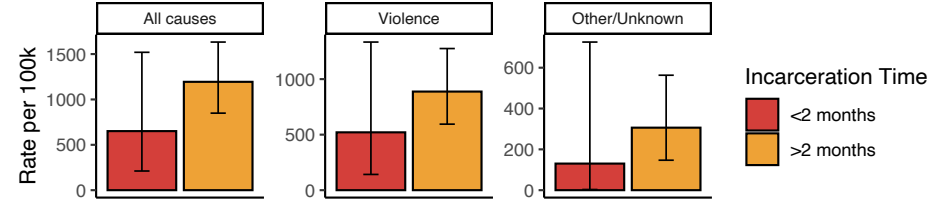

Supplement: S9 Fig — All-cause and cause-specific mortality rates per 100,000 person-years during and after the first 1 to 3 months of incarceration in each facility type. Rates were directly standardized to the age structure in the later period. Error bars indicate the 95% CI. IRRs between the earlier and later period were computed; significant differences are indicated (*p < 0.05; **p < 0.01). Causes for which there were fewer than 4 total deaths per time period were combined with other/unknown causes. 100k, 100,000 person-years; CI, confidence interval; comm diseases, communicable diseases; IRR, incidence rate ratio; non-comm diseases, noncommunicable diseases. (PDF) [file pmed.1003789.s013.pdf]

**A**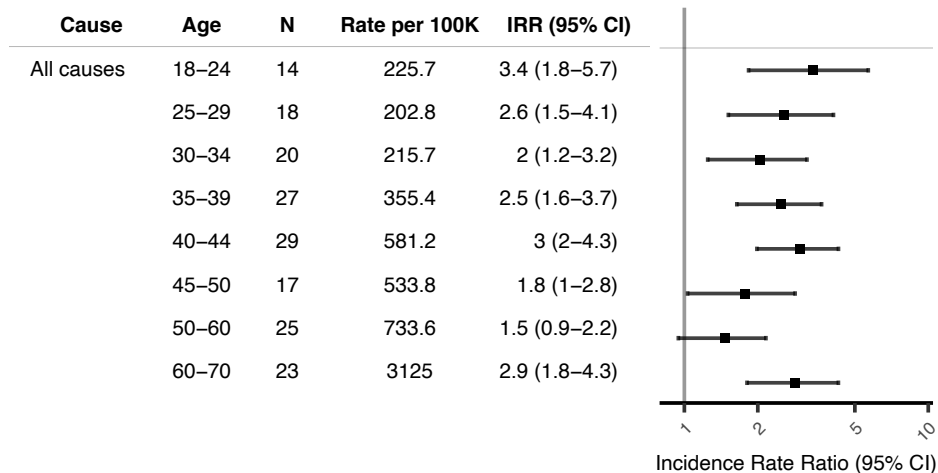**B**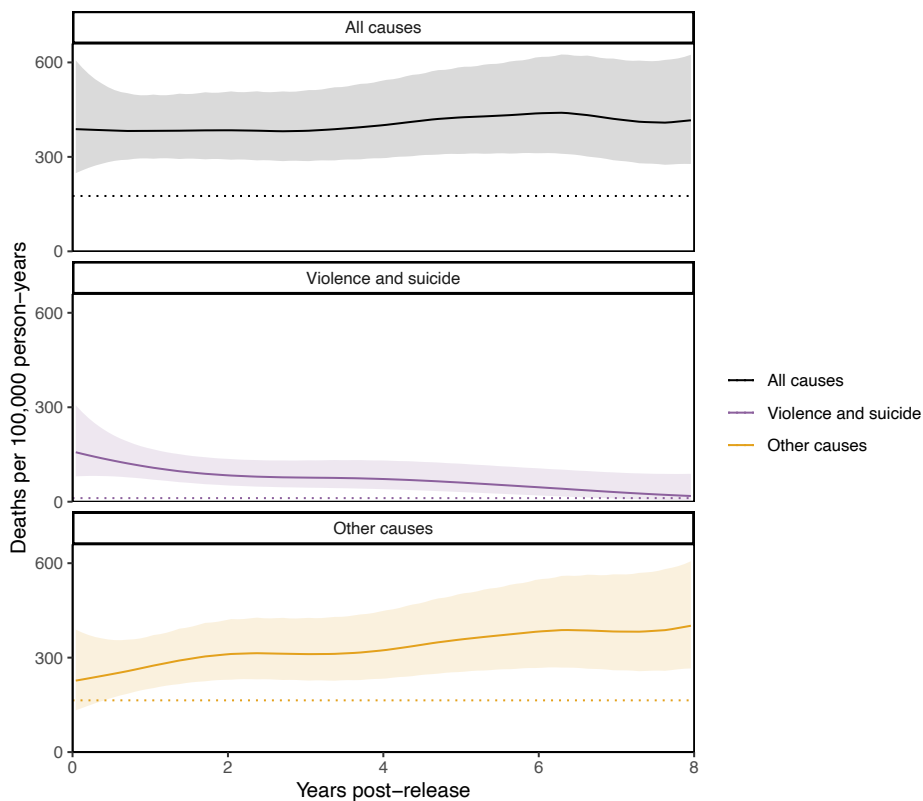

Supplement: S10 Fig — (A) Age-specific mortality rates for formerly incarcerated women. IRRs were computed relative to non-incarcerated female Mato Grosso do Sul residents. IRR, incidence rate ratio; N, number of deaths. (B) Instantaneous crude death rate among formerly incarcerated women for all causes of death (black), violence and suicide (purple), or causes other than violence and suicide (yellow). Bands indicate the 95% CI. Dotted horizontal line indicates the age-standardized mortality rate among non-incarcerated female residents, standardized to the age structure of the female formerly incarcerated population. 100K, 100,000 person-years; CI, confidence interval; IRR, incidence rate ratio; N, number of deaths. (PDF) [file pmed.1003789.s014.pdf]

**A**

Violence

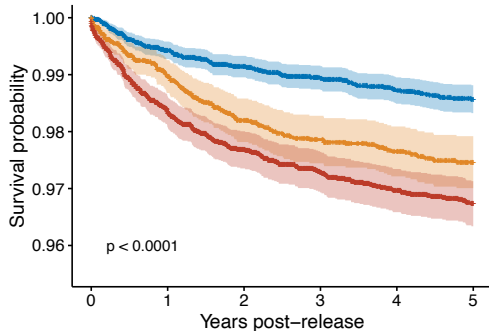

Other causes

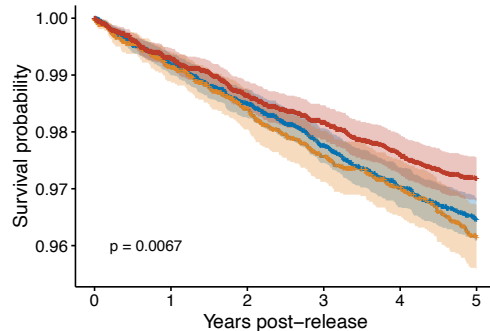**B**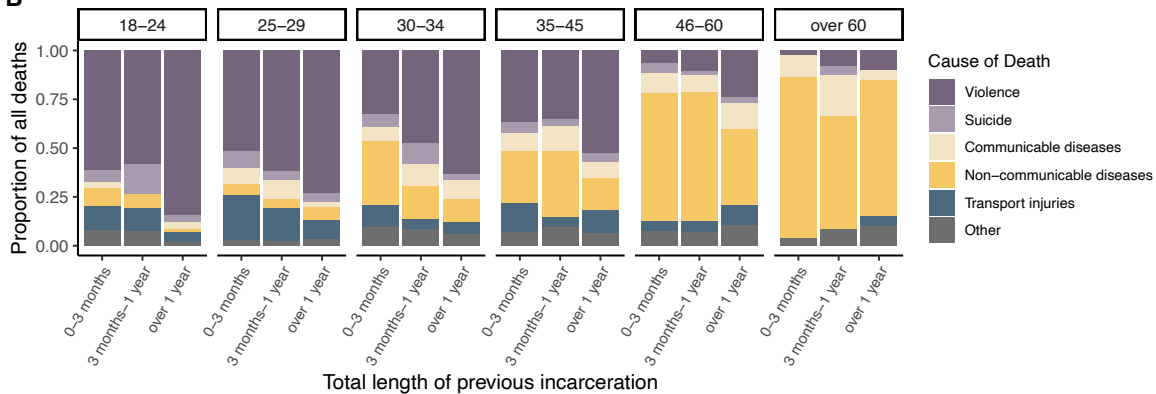**C**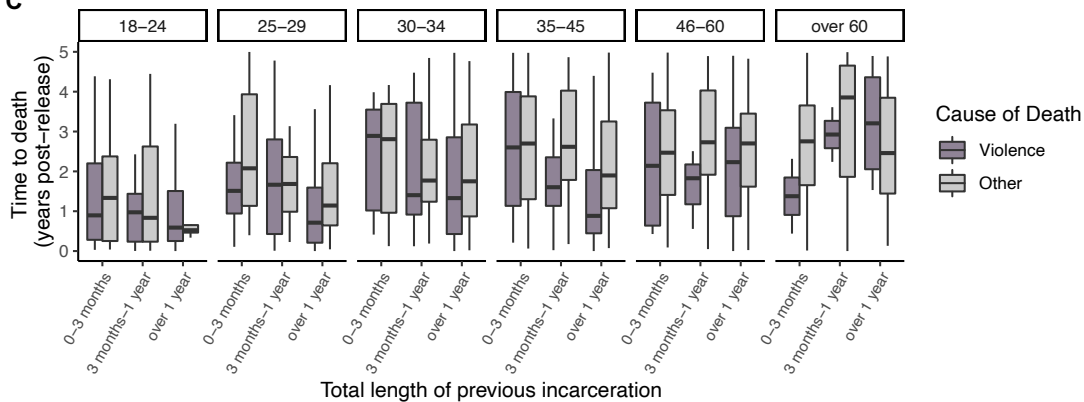

Supplement: S11 Fig — (A) Kaplan–Meier estimates of survival from violence or other causes for men post-release, stratified by total time incarcerated in any facility type. p-Values correspond to the log-rank test. (B) Stacked bar plots showing the association between total time incarcerated and proportions of violent deaths among men post-release, stratified by age group. (C) Boxplots depicting the association between total time incarcerated and post-release time to death (in years) for violent deaths and other causes, stratified by age group. All analyses in this figure were performed on a reduced cohort of men with consistent follow-up time (all censored at 5 years post-release). (PDF) [file pmed.1003789.s015.pdf]

**A**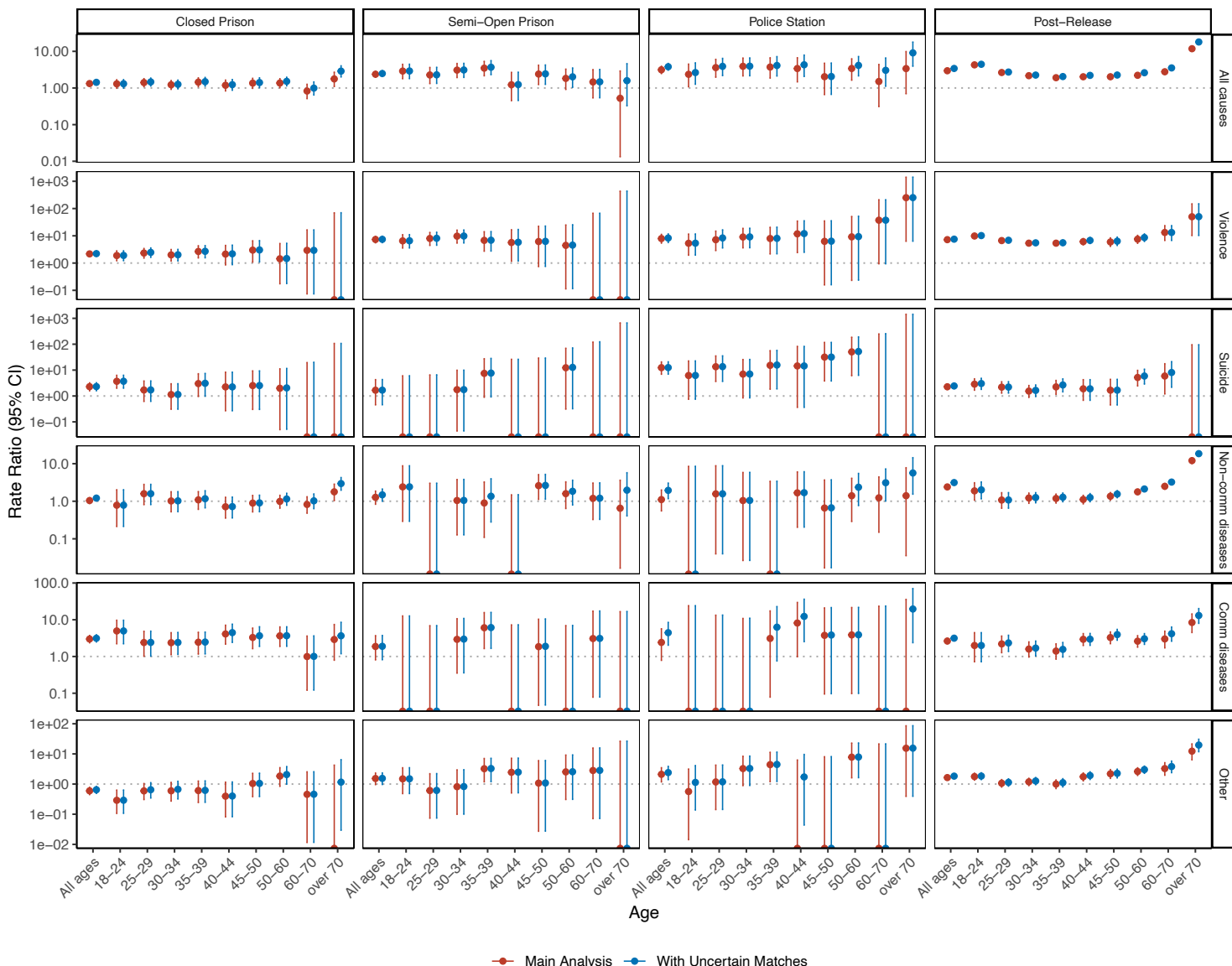**B**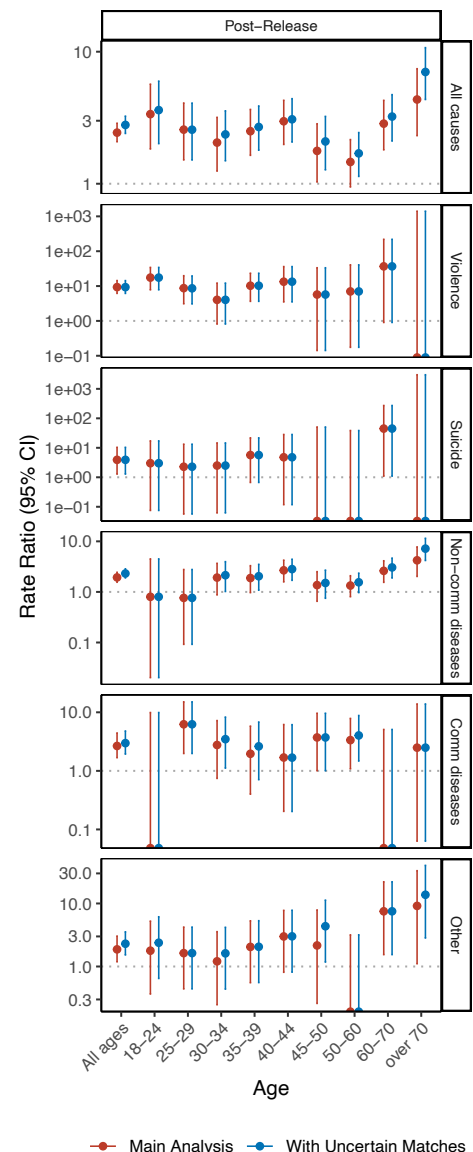

Supplement: S12 Fig — IRRs estimates for age-standardized and age-specific, all-cause and cause-specific mortality among (A) men and (B) women upon the exclusion (red) or inclusion (blue) of 345 matches for which mother’s name was missing and there were multiple perfect matches by name. Rate ratios are depicted on a log10 scale. 100k, 100,000 person-years; comm diseases, communicable diseases; IRR, incidence rate ratio; non-comm diseases, noncommunicable diseases. (PDF) [file pmed.1003789.s016.pdf]

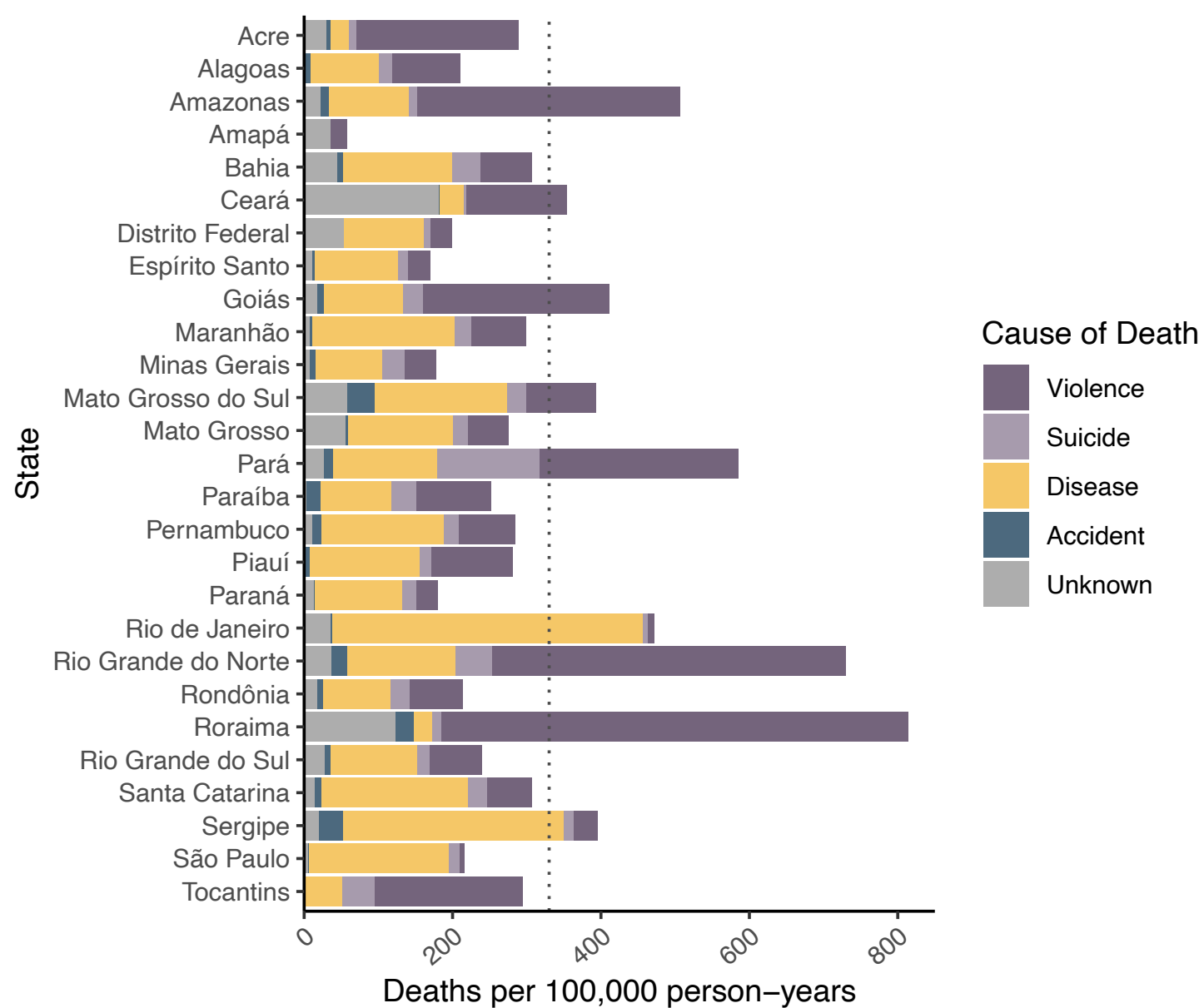

Supplement: S13 Fig — Bar plots depicting the crude mortality rate and proportions of cause-specific deaths during incarceration in each Brazilian state between 2016 and 2018, as reported by the Brazilian National Prison Department (DEPEN). The vertical dotted line indicates the mean crude mortality rate across all states. DEPEN, Departamento Penitenciário Nacional. (PDF) [file pmed.1003789.s017.pdf]
