# Supplementary material for: All-cause and cause-specific mortality during and following incarceration in Brazil: A retrospective cohort study
Source: PLoS Med. 2021 Sep 17;18(9):e1003789. doi: 10.1371/journal.pmed.1003789 (PMC8486113; doi:10.1371/journal.pmed.1003789)
Supplement: S5 Fig — Stacked bar plots indicating the proportions of deaths of women resulting from each cause category, stratified by age group and incarceration status. General population refers to female Mato Grosso do Sul residents who did not have a record in the state’s incarceration database between 2005 and 2018. Incarc, incarcerated. (PDF) [file pmed.1003789.s009.pdf]

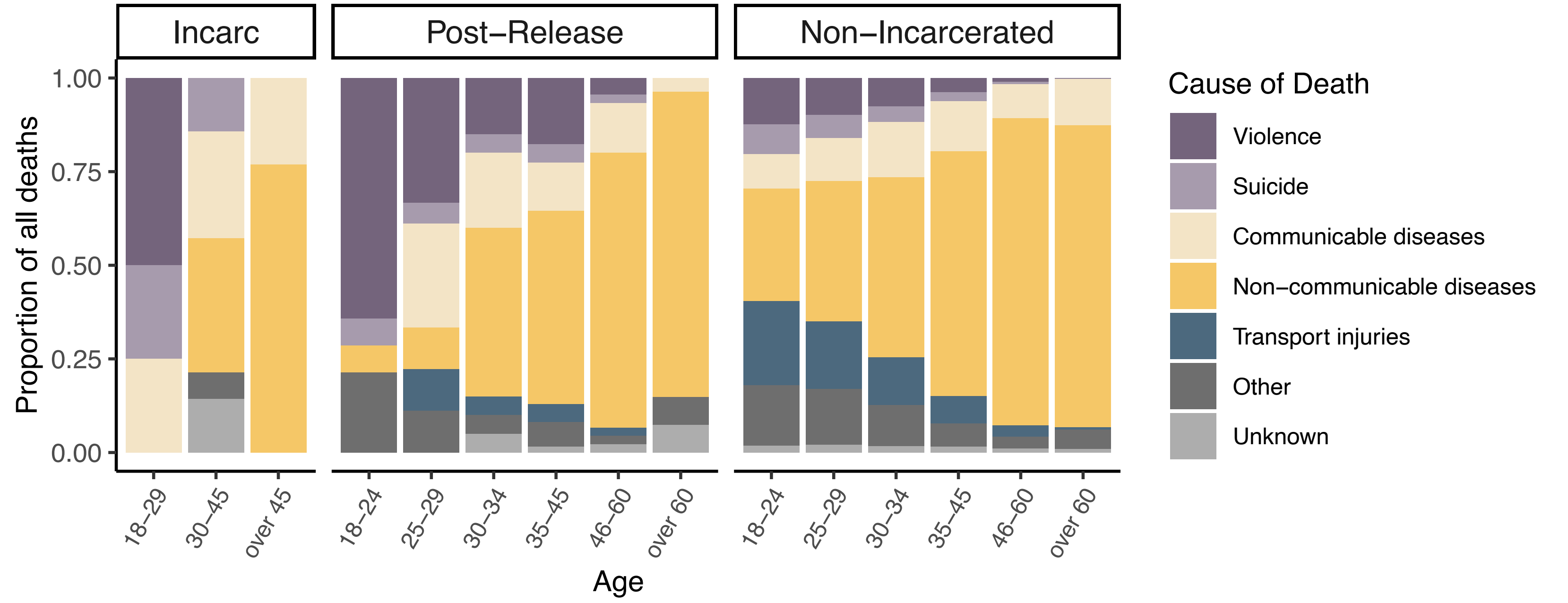
Incarc
Post−Release
Non−Incarcerated
Proportion of all deaths
1.00
0.75
0.50
0.25
0.00
18−29
30−45
over 45
18−24
25−29
30−34
35−45
46−60
over 60
Age
Cause of Death
Violence
Suicide
Communicable diseases
Non−communicable diseases
Transport injuries
Other
Unknown
