# Supplementary material for: All-cause and cause-specific mortality during and following incarceration in Brazil: A retrospective cohort study
Source: PLoS Med. 2021 Sep 17;18(9):e1003789. doi: 10.1371/journal.pmed.1003789 (PMC8486113; doi:10.1371/journal.pmed.1003789)

Semi–Open Prison

Cause	N	Rate per 100K	IRR (95% CI)
All causes	117	761.5	2.4 (2–2.8)
Violence	57	370.5	7.3 (5.5–9.5)
Suicide	4	26.1	1.7 (0.5–4.3)
Non–communicable diseases	26	169.5	1.3 (0.8–1.9)
Communicable diseases	8	52.2	1.9 (0.8–3.7)
Other	22	143.3	1.5 (1–2.3)

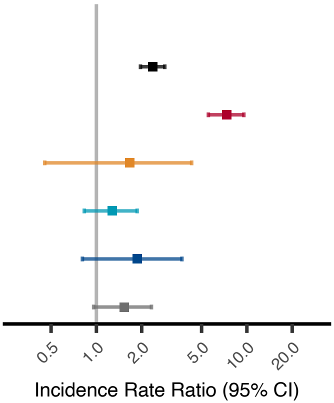

Police Station Lockup

Cause	N	Rate per 100K	IRR (95% CI)
All causes	77	1005.6	3.1 (2.5–3.9)
Violence	31	399.7	7.9 (5.4–11.3)
Suicide	15	194	12.4 (6.9–20.7)
Non–communicable diseases	11	147.6	1.1 (0.6–2)
Communicable diseases	5	67.3	2.4 (0.8–5.7)
Other	15	197	2.1 (1.2–3.5)

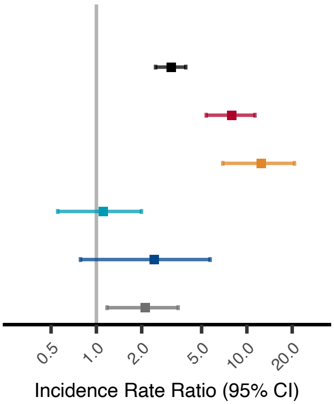

Youth Detention

Cause	N	Rate per 100K	IRR (95% CI)
All causes	45	1079.9	8.1 (5.9–10.8)
Violence	34	815.9	19.4 (13.3–27.5)
Suicide	2	48	2.9 (0.3–10.4)
Other	9	216	4 (1.8–7.6)

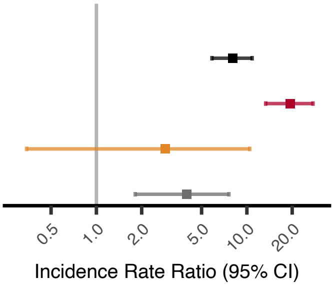

Supplement: S7 Fig — All-cause and cause-specific mortality rates per 100,000 person-years and IRRs for men and boys detained in semi-open prisons, police lockups, and youth detention. Rates were directly standardized to the age structure among incarcerated men. Crude, age-specific rates are depicted for boys 14 to 19 years of age in youth detention. IRRs were computed relative to non-incarcerated male Mato Grosso do Sul residents. A total of 6 deaths in semi-open prisons and 8 deaths in police stations were excluded due to missing age information. 100K, 100,000 person-years; IRR, incidence rate ratio; N, number of deaths. (PDF) [file pmed.1003789.s011.pdf]
