# Supplementary material for: All-cause and cause-specific mortality during and following incarceration in Brazil: A retrospective cohort study
Source: PLoS Med. 2021 Sep 17;18(9):e1003789. doi: 10.1371/journal.pmed.1003789 (PMC8486113; doi:10.1371/journal.pmed.1003789)
Supplement: Attachment — Submitted filename: mortalityPLOSMed_response_to_reviewers.docx [file pmed.1003789.s018.docx]

Note from the authors: line numbers in responses refer to the *clean* version of the manuscript (not the marked-up one).

**Requests from the editors:

Title: Please update the title to match PLOS Medicine’s typical format (see** [**https://journals.plos.org/plosmedicine/**](https://journals.plos.org/plosmedicine/) **for examples). ‘All-cause and cause-specific mortality during and following incarceration in Brazil: a registry linkage and modelling study’ or similar, for example.**
Response: We have revised the title to “All-cause and cause-specific mortality during and following incarceration in Brazil: a retrospective cohort study.”

**Abstract: Please add a final line to the ‘Methods and Findings’ section of the abstract that begins “The limitations of this study are” and summarises the main limitations. Please also ensure any conclusions are written in the past tense.**

Response: We have now added a final sentence to the “Methods and Findings” section of the abstract as follows (line 62): “The limitations of this study include inability to establish causality, uncertain reliability of data during incarceration, and underestimation of mortality rates due to imperfect database linkage.”

We also now ensure all conclusions are written in past tense.

**References: Please format these to remove and bold or italicised text and list the first six authors before ‘et al.’.**

Response: We have revised the formatting of the references to conform to this guidance.

**Please remove the ‘Author contributions’ section from the end of the manuscript as, in the event of publication, this information is presented in the metadata based on your responses to the submission form.**

Response: We have removed the “Author contributions” section from the end of the manuscript.

**If a protocol or statistical analysis plan were prepared in advance of the study, please can these be provided as supplementary materials and referenced appropriately in the methods (if an analysis plan was prepared, please highlight any analyses that were not pre-specified).**

Response: As this was a descriptive observational study, we did not have a pre-specified analytical plan.

**Please include an ‘Author Summary’ section as outlined here** [**https://journals.plos.org/plosmedicine/s/revising-your-manuscript**](https://journals.plos.org/plosmedicine/s/revising-your-manuscript)

Response: We have included an author summary as follows (line 92).

Why Was This Study Done?

- The global prison population is growing rapidly, particularly in Latin America
- Incarcerated and formerly incarcerated individuals may be at high risk of death from illness and external causes (i.e. homicide, suicide)
- Mortality during and after incarceration has been described in high-income countries but is poorly understood in low- and middle-income countries, including those in Latin America
- In Brazil, deaths during incarceration may be underreported, and there are no data on deaths after incarceration

What Did the Researchers Do and Find?

- We retrospectively followed over 114,000 individuals with recent incarceration in a Brazilian state and used database linkage to identify deaths between 2009 and 2018 occurring during or after incarceration
- We calculated rates of death from all causes and specific causes among incarcerated and formerly incarcerated individuals, and we compared them to those in the general population
- We found elevated mortality from violence, suicide, and communicable diseases during incarceration, which was underreported by national and state sources
- Following release, individuals were at increased risk of mortality from nearly all leading causes of death, with particularly high risk of violent death and suicide among men in the first two years post-release

What Do These Findings Mean?

- While we could not establish causality between incarceration and mortality, many of our findings indicate increased mortality risk associated with exposure to the carceral environment
- The exorbitant rates of violent deaths during and after incarceration point to deep-rooted social problems that cannot be solved by incarceration
- Accurate and comprehensive data on mortality during and after incarceration are needed in Brazil and other low- and middle-income countries where this issue remains poorly understood
- Structural interventions are needed to reduce the mortality risk in this population, including immediate improvement of conditions and healthcare within carceral facilities, supportive programs and services for the early transition period post-release, and development of alternatives to incarceration

**Please consider whether the term ‘sex’ would be more appropriate than ‘gender’ throughout the manuscript.**

As suggested, we have changed “gender” to “sex” throughout the manuscript.

**Please include line numbering in the margin.**
We have included line numbering in the margin.

**-----------------------------------------------------------
Comments from the reviewers:**

**Reviewer #1: This is a useful and well-conducted study on all-cause and cause-specific mortality during and following incarceration in Brazil. The study design, datasets, and statistical methods and analyses are mostly adequate. However, there are still a few issues needing attention.**

Response: We thank the reviewer for their positive feedback and for their suggestions on how to improve the manuscript.

**1) It said all rates were age-standardised, but why not both age and sex standardised as there were female prisoners too?**

Response 1: We recognize that the omission of age-standardized rates for incarcerated women may have been confusing and appreciate the reviewer pointing this out. Originally, we excluded this analysis due to the relatively sparse number of deaths. However, we have considered the reviewer’s comment and now include age-standardized rates for incarcerated women in any facility type (Figure 3). These findings are now described in the following paragraph of the results section (line 399):

## “Mortality rates among incarcerated women

For incarcerated women, the number of deaths was too small to perform stratified analyses by facility type; we therefore combined deaths across facility types. The all-cause age-standardized mortality rate among incarcerated women in any facility type was 268 (95% CI, 182-380) per 100,000 person-years, comparable to women without recent incarceration (291 per 100,000; 95% CI, 285-296) (Fig. 4). While incarcerated women were less likely to die from non-communicable diseases (IRR = 0.6, 95% CI, 0.3-1), they were at increased risk of death from suicide (IRR = 6, 95% CI 1.2-17.7) and communicable diseases (IRR = 2.5, 95% CI 1.1-5.0).”

**2) In statistical analysis section, it says "Deaths of individuals under 18 years of age were excluded from rates analysis due to missing age structure information for those under 18". As the main analyses were on rates, does this mean the study cohort should be defined as those over18 years old? It's a bit confusing throughout the paper, in text, tables and figures that some are on 18+, some on 15+ and some on 16+?**

Response 2: We originally wanted to include as many deaths as possible; therefore, in the analyses of cause-of-death proportions and instantaneous hazard, we had included deaths of those under 18. We agree with the reviewer that the varying minimum age in different analyses may have been confusing. We did not have individual-level age in SIGO to exclude those under 18 from the cohort, but we now exclude deaths of those under 18 years old from all analyses for clarity and consistency, with the exception of deaths in youth detention (ages 14-19). We have now fixed age group labels and updated numbers of deaths in Figures 1-2, 5, S2-S5, S8, and Table S2, and we have fixed the sentence identified by the reviewer as follows (line 297):

"We excluded deaths of individuals under 18 years of age in closed prisons, semi-open prisons, police stations, and post-release due to missing age structure information for those under 18. However, we were unable to exclude individuals under 18 from the study cohort due to lack of individual-level age information in SIGO. All deaths in youth detention were included (ages 14-19), from which we calculated crude rates in the absence of age structure information."

**3) There is no table at all in the main text. Need a comprehensive and informative table 1 on summary of the dataset on baselines (age, sex, and etc) and possibly outcomes of interests.**

Response 3: We appreciate the reviewer’s suggestion to add a main table describing the baseline characteristics and outcomes of the cohort. We have now added the following as Table 1, which describes the number of individuals with one or more intervals in each setting, summary statistics for interval duration in each setting, the age structure of the incarcerated population, and the breakdown of deaths by age, all stratified by sex.

**4) Regarding figures, Figure 2 can be moved to supplementary as mainly details on data linkage. Figure 4 presents the main results of the paper but why there was no "closed prison (women)"?**

Response 4: We have moved Figure 2 to supplemental information as suggested by the reviewer. As mentioned in response 1, we now include mortality rates among incarcerated women (Figure 3). As there were only 31 deaths among incarcerated women across all carceral facility types (closed prisons, semi-open prisons, police stations), we examined these altogether. We now provide an explanation for this in the results paragraph on mortality among incarcerated women (line 400):

“For incarcerated women, the number of deaths was too small to perform stratified analyses by facility type; we therefore combined deaths across facility types.”

**5) The deaths trend analysis after release as shown in Figure 5 is very interesting but lacks of solid interpretation on the reasons behind the trend which should be supported by the literature and statistics of trends in risk factors.**

We appreciate the opportunity to provide interpretation and references to explain the trends in mortality following release as shown in Fig. 5. We added an analysis of the association between early violent death and cumulative incarceration time in response to another reviewer (Fig. S11) and have added the following commentary to the discussion section (line 508):

“Following release, all-cause mortality was elevated among men and women of all ages for nearly all leading causes of death, with particularly high violent death and suicide rates in the early period that declined sharply in the first two years post-release. Among men, younger age and increasing cumulative incarceration time were associated with early violent death post-release. Our findings mirror trends in high-income countries [13, 16, 48], where younger age, male sex, length of incarceration, and/or repeated incarceration were also risk factors for early post-release mortality [8, 13]; however, in contrast to studies in high-income countries [11, 15], drug overdose only comprised a small fraction of post-release deaths in our cohort. Therefore, additional research is needed to identify risk factors for post-release mortality in settings with different social conditions and leading causes of death [48].”

**6) In conclusions in the abstract, it says "The cause-specific trends and risk of incarceration-associated mortality in Brazil differ from those reported in high-income countries...". However, this is not a comparative study, and can't compare like for like, therefore it's not adequate to conclude this as a key conclusion of the study.**

Response 6: The reviewer is correct that this was not a comparative study. We have now removed this statement from the conclusions in the abstract.

**Reviewer #2: "All-cause and cause-specific mortality during and following incarceration in Brazil."
This is an interesting and relevant study examining mortality rates among criminal justice-involved populations in the Brazilian state, Mato Grosso do Sul. The authors find elevated mortality rates among this population, with particularly alarming rates of death due to violence both while under supervision and post-release.**

Response: We appreciate the reviewer’s positive comments and constructive feedback for improving the framing and discussion.

**1.      My main issues with the manuscript as it stands lie in the framing of the study and the discussion of findings.
a.      With regard to the former, the paper is framed as a study of "incarceration-associated mortality" (see abstract and introduction). However, this is a misnomer in a couple of ways. For one, the paper does not and cannot trace deaths to being caused by or related to incarceration itself. These are death rates among justice-involved persons- NOT incarceration-associated mortality. Second, the study also includes mortality rates of those in police stations. Is this being defined as incarceration as well? Please clarify.**

Response 1a: We agree with the reviewer that the framing and language used in the paper is of utmost importance and recognize the reviewer’s point about avoiding phrases such as “incarceration-associated mortality” that may imply causality. We have now replaced this phrase with, “mortality among currently and formerly incarcerated individuals” or “mortality during and after incarceration.”

We also recognize the insufficient clarity around detention in police stations. Indeed, we define detention in police stations as incarceration. In Brazil, police stations often serve as de facto detention facilities due to severe overcrowding in many prisons. In the Introduction we clarify (line 163): “Approximately 5% of the incarcerated population are detained in police stations [19]; while these facilities are designed for short-term detention of at most a few days, some have reported detentions of months or years in these settings [21, 22].” We have now included additional clarification in Methods (line 235): “We include detention in police stations as a form of incarceration, as some individuals are detained in police stations for extended periods pre-trial and post-sentencing due to severe prison overcrowding [21, 22].” We also have added a Table 1 that describes median and interquartile range of duration in each facility type. A section of the table is pasted below and shows that 25% of men and women in police stations are detained for longer than 17 and 21 days, respectively.

|  | | **Men and boys (N=105,465)** | **Women (N=9,286)** |
| --- | --- | --- | --- |
| **Median duration in days (IQR)** | Closed prisons | 153 (21-473) | 184 (34-482) |
|  | Semi-open prisons | 62 (13-169) | 93 (29-240) |
|  | Police stations | 3 (1-17) | 2 (1-21) |
|  | Youth detention | 229 (27-758) | 300 (46-498) |
|  | Post-release | 348 (50-1300) | 849 (131-2262) |

**b.      As for the latter, and this is a similar issue to the one abovementioned, the discussion makes problematic statements, but most notably:
"These disparities [in mortality] may be partially explained by pre-existing vulnerabilities in system-impacted populations, but likely also reflect the long-term negative effects of incarceration due to communicable diseases, chronic stress, social deprivation, malnutrition, and/or medical neglect, as well as the social conditions associated with previous incarceration, such as stigma, poverty, and barriers to housing, healthcare and employment."
 First, the author cannot establish causality between exposure to carceral settings and death, or causes of death. Second, the study does not speaks to this swath of potential hypotheses and variables. Third, things like communicable diseases were actually pretty comparable across post-release and general populations in the findings.
Importantly, the authors should acknowledge that they cannot establish causality, let alone speculate about underlying reasons for causality. Also, justice-involved populations exhibit riskier lifestyles, including violent behavior, gang involvement, and substance use - all of which may contribute to these disparities in mortality.**

Response 1b: We acknowledge the reviewer’s concerns about the use of causal language to describe the results of this study, which did not investigate causality. We have added the following to the limitations to explicitly acknowledge that we cannot establish causality (line 542): “First, in the absence of a counterfactual, we were unable to establish causality between exposure to incarceration and the mortality trends described. Insufficient data also precluded our ability to adjust for socioeconomic factors, pre-trial status, conviction type, or other potential confounders.”

Regarding the specific statement that the reviewer identified, we have revised the commentary as follows (line 518):

“Upon release, formerly incarcerated men and women also experienced elevated disease mortality that further increased over time. This disparity was a result of increased communicable and non-communicable disease mortality post-release and corresponds to studies reporting high incidence of infectious diseases [49, 50] and chronic medical conditions [51] among formerly incarcerated individuals. Recent work in Mato Grosso do Sul found bacterial genomic evidence of new tuberculosis diagnoses among formerly incarcerated individuals that could be traced to transmission within carceral facilities [52], indicating that elevated communicable disease mortality post-release may at least be partially attributable to incarceration. However, more data are needed to determine the respective contribution of incarceration versus pre-existing vulnerabilities to disease mortality post-release.”

**2.      The causes of death while incarcerated piqued my curiosity in terms of the validity and reliability of these data. It strikes me that prison administrators and criminal justice organizations might have incentive to manipulate the data in this case - for example, claiming a death was a suicide rather than due to violence perpetrated by another prisoner or by a guard. Can the authors speak a little bit more about whether there might be reliability or validity issues of this nature?**

## Response 2: We agree that it is important to consider the reliability of cause-of-death data from inside carceral facilities, and in particular we agree with the reviewer’s concern that homicides might be misreported as suicides. We state in the discussion section, referring to the exorbitant suicide rates in police detention (line 489), “However, given potential underreporting of violent deaths in Brazil [44]; it is possible that some violent deaths were incorrectly classified as suicide, particularly in settings where administrative oversight may be limited.”

## Per the reviewer’s suggestion, we have now added a sentence to the limitations addressing this issue (line 545): “Second, cause-of-death classifications during incarceration may have reduced reliability, as evidenced by the greater proportion of deaths of unknown or imprecise cause among incarcerated individuals compared with non-incarcerated individuals (Fig. 3).”

**3.      I would appreciate more discussion of the differences between data culled from DEPEN vs. SIGO. By that I do not mean how well records were matched, but more so - do these agencies have different missions, responsibilities, how do they collect these data similarly/differently?**

Response 3: We agree with the reviewer that considering these factors is important for interpreting the databases’ mutual inconsistency and underreporting of deaths. We have therefore included more information on the two databases in Methods (line 201):

“Maintained by police in Mato Grosso do Sul, SIGO is a state database of all individual-level movements (i.e. arrests, court appearances, transfers, releases, and deaths) within the state’s criminal legal system, including movements through police stations, prisons, and youth detention…

We also retrieved publicly available, aggregate data from the national DEPEN information system [20] on prison deaths by state; in Mato Grosso do Sul this information is entered by the state prison administration (AGEPEN).”

We have also added the following to the discussion (line 501):

“Finally, we found that deaths in custody were underreported by and inconsistent between national (DEPEN) and state (SIGO) sources. These discrepancies were not explained by the lack of DEPEN data on deaths in police stations or youth detention but may instead indicate differences in mission, operations, and management between the agencies responsible for data reporting (state police for SIGO, state prison administration for DEPEN).”

**4.      It strikes me that this research runs counter to some work on United States prison populations, which finds that being in prison can be protective for some populations in terms of mortality. This might be worth a discussion. For example, please see the work of Chris Wildeman and Evelyn Patterson.**

Response 4: We thank the reviewer for commenting on this interesting difference between our findings in Brazil and some reports from US prisons. We have added the following commentary along with citations to the work of Chris Wildeman and Evelyn Patterson (line 476):

“Incarcerated men experienced heightened risk of all-cause mortality across all facility types and nearly all age groups, driven by elevated rates of death from violence, suicide, and communicable diseases. While these findings were consistent with some studies from high-income countries that reported increased risk of homicide [8, 38], suicide [39-41], and infectious disease deaths [6, 10] during incarceration, they contrast with other studies that reported reduced all-cause mortality and homicide during incarceration [5], particularly among subgroups with higher baseline mortality [9, 10, 42, 43]. The root causes of these differences are undoubtedly complex and were not addressed by the present study but may include varying prison conditions, management, and culture.”

**5.      In general, the findings section was hard to follow and seemed unstructured/disorganized. I recommend cutting back on the amount of acronyms used and to organize the discussion of findings in order of interesting or meaningful comparisons. For example, a paragraph about gender differences, a paragraph about differences across recently vs. not recently incarcerated persons, a paragraph about differences across facility types, etc.**

Response 5: We appreciate the reviewer’s suggestions for improving the structure and organization of the findings. As suggested, we have made substantial changes to the organization to improve readability. We broadly partitioned the findings into mortality during incarceration and mortality post-release. We added subheadings in both the results and discussion sections to orient the reader, and organized the discussion section similarly to how the reviewer suggested:

In Results:

## Linkage of incarceration and mortality databases reveals deaths during detention and following release

## Leading causes of death varied by age, sex, incarceration status, and facility type

## Mortality rates among incarcerated men in closed prisons

## Mortality rates among incarcerated men and boys in other facility types

## Mortality rates among incarcerated women

- Mortality rates among formerly incarcerated men and women

In Discussion:

- Summary of findings
- Mortality during incarceration
  - Paragraph 1: discussion of mortality among incarcerated men
  - Paragraph 2: differences by facility type
  - Paragraph 3: discussion of mortality among incarcerated women
  - Paragraph 4: discrepancies with reporting sources
- Mortality post-release
  - Paragraph 1: time trends and violent mortality
  - Paragraph 2: disease mortality
- Implications
- Limitations
- Conclusions

Regarding usage of acronyms, we have removed all instances of “ASMR” (age-standardized mortality rate); reduced usage of “IRR” (incidence rate ratio) in the text; and re-defined DEPEN, SIGO, and SIM in each results or discussion paragraph that mentioned them.

**6.      The authors should not claim that their findings from one state in Brazil could be generalizable (see conclusion). This is simply incorrect from a methodological standpoint. Also, bolstering this claim by saying that the mortality rates in prison are similar to other places in Brazil is a misleading justification of this point.**

Response 6: We acknowledge the reviewer’s point and have removed this phrase from the conclusion in the abstract. We do feel it is important context to show that mortality in prisons in Mato Grosso do Sul is not an outlier compared to other states in Brazil, but we agree that this should not be described as generalizing our findings. We acknowledged this in the limitations, where we now present a condensed and modified version of the comparison between Mato Grosso do Sul and other Brazilian states (line 557):

“Finally, our findings may not be generalizable to other states or countries. However, Mato Grosso do Sul is likely not an outlier in Brazil: the crude mortality rate during incarceration in Mato Grosso do Sul, as reported by DEPEN, was similar to the mean national rate (Fig. S13).”

**Reviewer #3: The following paper is ambitious in its goal to provide overall and cause-specific estimates of death rates during and following incarceration in Brazil. These estimates were compared to non-incarcerated residents Nevertheless, by linking the national mortality database and prison records, the authors found that deaths in custody were more than twice as high as deaths reported. While I commend the authors for breadth of the work, I found myself confused at times, regarding which populations were included or excluded from the analyses. Further, the methodology utilized to undertake the analysis requires much more detail than provided in the manuscript. To this end, citations would be helpful. My general recommendation is that the authors focus on mortality during incarceration (or after incarceration), but not both in the same paper. I found it overwhelming, and limiting it to such would allow the authors to dedicate more space to providing details regarding the methodology and underlying assumptions. Regardless, the findings add to this literature and I did think the results of the mortality levels during and after incarceration increase the knowledge to the general area. I enumerate my specific concerns regarding the manuscript below.**

Response: We appreciate the reviewer’s summary of our work and positive comments on the breadth and contribution of these findings. We acknowledge that the presentation of all of these findings, from men and women across different facility types during and post-incarceration, was dense and difficult to present in a clear manner. At the same time, given the increased risk of mortality during and post-incarceration and the important differences in causes across these periods, we feel it is important to present these results together to describe health outcomes across the carceral interaction. As such, we have incorporated the reviewer’s feedback and made substantial changes to better organize and clarify our methods and findings, as described in greater detail in response to each point below.

**1.      Describe how you projected the formerly incarcerated population in more detail. Right now, I understand that the authors stratified person-years by release year of the cohort, but I do not know anything about how or why a person exited the formerly incarcerated population.**

Response 1: We thank the reviewer for pointing out the lack of clarity around how we censored formerly incarcerated individuals. Indeed, for all analyses (including cumulative rates, instantaneous hazard, and survival), we censored formerly incarcerated individuals upon reincarceration in any facility type, death, or the end of the study period. These censored intervals were then combined to obtain the cumulative person-time in the formerly incarcerated population, stratified by year of release and years elapsed post-release.

## To improve clarity, we now describe censoring separately for intervals during incarceration and post-release (they were previously combined in one sentence) (line 223):

“Intervals of incarceration in each facility type were censored upon transfer to a different facility type, release, death, or the end of the study period. Exceedingly long intervals in police stations, semi-open prisons, and youth detention were truncated to account for underreporting of releases from these facilities (see Appendix). Intervals in the community following release from prison (closed or semi-open) were censored upon reincarceration in any facility type, death, or the end of the study period.”

## We additionally restructured the methods, splitting the methods for person-time and age-structure estimation into separate sections for the incarcerated population, formerly incarcerated population, and population without recent incarceration.

**2.      Relatedly, it is not clear how the authors distinguished formerly incarcerated individuals with recent incarceration from the comparison group of the general population.**

Response 2: We appreciate the reviewer pointing out that this distinction was not clear. We have clarified this in two places in the methods as follows:

Pre-processing of SIGO incarceration database (line 218):

“Ultimately, we identified 143,551 unique individuals with at least one record in SIGO between 2005 and 2018; these individuals were excluded from the general population to obtain the population without recent incarceration history.”

Person-time and age structure of the non-incarcerated population (line 284)

“For the population without recent incarceration history (hereafter referred to as the “non-incarcerated population”), we used projected binned age structure for Mato Grosso do Sul from the Brazilian Institute of Geography and Statistics (IBGE) [33] and estimated cumulative age-specific person-time using the same method as described for the incarcerated population. We excluded individuals with any record in SIGO from the non-incarcerated population by subtracting person-time of the incarcerated and formerly incarcerated population, the latter of which was re-computed using releases from any facility type (not only releases from prison).”

**3.      Please explain more details regarding the estimation of the age structure.**

Response 3: We appreciate the opportunity to provide more details about estimating the age structure, as this was important to the calculation of age-standardized rates. In the main text, we now cite the packages used and refer readers to the appendix for more details (line 262):

“To obtain more granular estimates of age structure, we fit truncated negative binomial distributions to the binned age data **using the R packages *fitdistrplus, truncdist,* and *revengc* [31-33] (see Appendix)**, with separate distributions by sex and calendar year.”

In the appendix, we now provide the following detailed explanation of the estimation of age structure (line 124):

**“Estimation of age structure for incarcerated population and population without recent incarceration**

We started with age structure data reported in wide bins that were not directly comparable between the incarcerated population and the population without recent incarceration. To enable more precise calculation and comparison of age-specific and age-standardized rates between populations, we fit truncated negative binomial distributions to the binned data using maximum likelihood estimation, implemented through the *fitdistcens* function from the R package *fitdistrplus* and adapted for truncated distributions using the *truncdist* package. We estimated starting mean and dispersion values using the *revengc* package. We compared the fitted distributions to the binned data and re-ran the estimation procedure with adjusted weights for certain age groups in order to improve the fit. For instance, we observed that the initial distribution underestimated the proportion of those in the 25-29 age group, which was also the most populous according to DEPEN in most years; we therefore set that age group to have twice the weight of other age groups in the estimation procedure. To further improve the consistency of our estimates with reported age structure, we adjusted the estimated counts to correspond proportionally to the reported binned data. For example, if the age structure was reported such that the 25-29 age group comprised 25% of the incarcerated population in 2012, we scaled our modeled counts for each year in the 25-29 age group to sum to 25% of the total incarcerated person-time in 2012. This way, we utilized the shape within each age bin, as derived from the fitted distribution, while retaining the reported proportions within each age group. For reported age bins that were extremely wide (i.e. age 46-60), this enabled more granular, accurate comparison of mortality rates across populations with starkly different age compositions.”

**4.      For the formerly incarcerated persons, the population only seems to change over time when persons die. However, there is certainly the risk of reincarceration. You mention this as means for individuals to be censored in the Pre-processing of SIGO incarceration database section, but it is not clear. I am guessing that the prison data contained this information at the individual level, however, my understanding is that you used summary level data. Please help me understand.**

Response 4: We did use individual-level reincarceration data from SIGO to censor post-release intervals upon reincarceration. We describe ways in which we clarified this in our response to point 1.

**5.      The differences in causes of death by facility make me question the assumption regarding each facility having the same age structure. Is there another option? That is, do you have wide age bins for each facility type?**

Response 5: We acknowledge the reviewer’s point about how differences in age structure by facility type may have affected estimated rates. Unfortunately, DEPEN only reported age structure for prisons (closed and semi-open combined), and there were no data on age structure in police stations. Furthermore, SIGO did not contain individual-level age information. We have now elaborated on the sentence mentioning this limitation in the discussion (line 552): “Next, we did not have facility type-specific age structure information; consequently, our assumption of the same age structure across facility types may have affected rate estimates.”

**6.      I noted above that at times I got lost regarding the population included or excluded in analyses. I think this can easily be solved if the authors reiterate the population included/excluded. It would also be helpful, if the authors decide to keep both analyses, to partition the details of each piece in the data & methods and results sections. For example, describe the data and methods for the estimation of rates during incarceration, and then separately describe the data and methods for the post-incarceration mortality. It may be that the majority of my concerns can be taken care of by doing this.**

Response 6: We acknowledge it can be challenging to keep track of all the different analyses and populations included/excluded. As mentioned in response to point 1, we restructured the methods section to describe the methods separately for each population. We also partitioned the results and discussion sections, as the reviewer suggested, into mortality during incarceration and mortality post-release. We split up the figure of age-standardized rates into one main figure for rates during incarceration (Fig 3) and one for rates post-release (Fig 4). We further improved organization and clarity by adding subheadings as follows:

In Results:

## Linkage of incarceration and mortality databases reveals deaths during detention and following release

## Leading causes of death varied by age, sex, incarceration status, and facility type

## Mortality rates among incarcerated men in closed prisons

## Mortality rates among incarcerated men and boys in other facility types

## Mortality rates among incarcerated women

- Mortality rates among formerly incarcerated men and women

In Discussion:

- Mortality during incarceration
- Mortality post-release
- Implications
- Limitations
- Conclusions

Finally, we added a supplementary table (Table S1, pasted below) which lists the analyses for each population, the reference population used for rate ratios, and the corresponding figures.

**7.      I keep coming back to the decision to censor individuals upon movement to a different facility. It makes sense when thinking about facility specific death rates. However, part of the argument regarding deaths relates to the exposure to the settings, which makes me wonder about cumulative exposure in correctional settings. Further, one of the issues above regarding the formerly incarcerated population becomes less clear. For each population, does the movement censor the observation? It cannot because there are the release cohorts. I think I just need clearer depiction in the text.**

Response 7: We agree with the reviewer’s interest in the effects of cumulative exposure to incarceration on mortality. To investigate this, we performed a few additional analyses to examine the association between cumulative incarceration time and mortality post-release and include a new figure (Fig S11). We describe these analyses in Methods (line 311):

“To analyze the association between total length of incarceration and mortality post-release, we generated a reduced cohort of men whose last recorded release from prison was at least five years before the end of the study period (N=23,608). Post-release intervals longer than five years were censored at five years to obtain consistent periods of observation. We computed each individual’s cumulative time incarcerated in any facility type prior to release and estimated survival from violent death or other causes using Kaplan-Meier, with log-rank tests for significance (*survival* package in R [36]). While we were unable to perform age-adjusted survival analysis due to lack of individual-level age information in SIGO, we examined within-age-group associations of cumulative incarceration time with cause-of-death and time-to-death post-release.”

We report our results as follows (line 428):

“Younger age was associated with greater proportions of violent deaths and shorter time-to-death post-release (**Fig S11)**. Furthermore, among men under age 46, increasing cumulative time incarcerated in any facility type was associated, across age groups, with increased odds of early death from violence and other causes following release.”

We discuss these findings as follows, along with commentary on risk factors for post-release mortality as requested by another reviewer (line 510):

“Following release, all-cause mortality was elevated among men and women of all ages for nearly all leading causes of death, with particularly high violent death and suicide rates in the early period that declined sharply in the first two years post-release. Among men, younger age and increasing cumulative incarceration time were associated with early post-release death, especially from violence. Our findings mirror trends in high-income countries [13, 16, 48], where younger age, male sex, length of incarceration, and/or repeated incarceration were also risk factors for early post-release mortality [8, 13]; however, in contrast to studies in high-income countries [11, 15], drug overdose only comprised a small fraction of post-release deaths in our cohort. Therefore, additional research is needed to identify risk factors for post-release mortality in settings with different social conditions and leading causes of death.”

Regarding the reviewer’s question about censoring, we have provided additional clarification in our response to point 1. We also defined a new section in methods, “Identification of study cohort,” where we now explicitly state (line 237), “For individuals with multiple incarcerations and/or releases, all intervals during the study period were included.”

**8.      In the abstract, the authors use the term "excess incarceration-associated mortality". When I read the term excess with mortality, I immediately think of deaths in excess of the expected. I do not think the authors seek to communicate such primarily because they do not do such, my understanding is that their study sets the foundation for future work. That is, this study serves as a baseline. I understand that the death rates found exceed that reported by the government, but as the authors also state, this is due to underreporting and insufficient data.**

Response 8: We appreciate the reviewer’s point about the lack of clarity with the use of the word “excess” in this context. We have removed all instances of the word throughout the manuscript. In response to a comment from reviewer 2, we also added the following to the limitations to explicitly acknowledge that we cannot establish causality (line 542): “First, in the absence of a counterfactual, we were unable to establish causality between exposure to incarceration and the mortality trends described. Insufficient data also precluded our ability to adjust for socioeconomic factors, pre-trial status, conviction type, or other potential confounders.”
