# Supplementary material for: All-cause and cause-specific mortality during and following incarceration in Brazil: A retrospective cohort study
Source: PLoS Med. 2021 Sep 17;18(9):e1003789. doi: 10.1371/journal.pmed.1003789 (PMC8486113; doi:10.1371/journal.pmed.1003789)
Supplement: Attachment — Submitted filename: mortalityPLOSMed_response2.docx [file pmed.1003789.s019.docx]

**Requests from Editors:**

**Please state that your study did not have a pre-specified analysis plan in the methods section.** We have now included this statement in the Methods (line 224): “We did not have a pre-specified analysis plan.”

**Please include your study design (retrospective cohort study) in both your abstract and methods, as it currently only appears in your title and your conclusions.**

We have added this to the abstract (line 50): “We linked incarceration and mortality databases for the Brazilian state of Mato Grosso do Sul to obtain a **retrospective** cohort of 114,751 individuals with recent incarceration.”

We have added this to the methods (line 200): “We carried out a retrospective cohort study on mortality during and following incarceration in Mato Grosso do Sul state by linking the mortality registry with the state’s incarceration database.”

**Line 45: The final line of the background section of your abstract should clearly state the study question. Please add an additional line to this effect.** We modified the last sentence as follows: “In Brazil, insufficient data have precluded our understanding of all-cause and cause-specific mortality during and after incarceration.”

**Line 114: Please remove the second bullet point in the ‘What do these findings mean?’ section of your author summary, as I feel it is too broad a statement to be included here and is better placed in the discussion where a more nuanced approach can be taken.** We have removed this bullet point.

**Line 566: Please include ‘to our knowledge’ whenever making claims of primacy (‘As the first systematic study’).** We have added this phrase to line 599 as follows: “As the first systematic study, to our knowledge, of mortality during and after incarceration in Latin America and in LMIC more broadly…”

**Figure S9: Please define what your error bars show in the figure legend.** We have added to the figure legend (line 689): “Error bars indicate the 95% confidence interval.”

**References 20, 28, and 35: Insufficient material is presented to allow the reader to locate these sources – please provide more detail and cite using Vancouver style (see guidelines here:** [**https://journals.plos.org/plosmedicine/s/submission-guidelines#loc-references**](https://journals.plos.org/plosmedicine/s/submission-guidelines#loc-references)**)**

We have edited these citations to include URLs as follows:

20. Human Rights Watch. Behind Bars in Brazil. 1998 [cited 2021 August 28]. Available from: https://www.hrw.org/sites/default/files/related_material/BRAZL98D.pdf.

28. SeatGeek. fuzzywuzzy. GitHub; 2017 [cited 2021 August 28]. Available from: https://github.com/seatgeek/fuzzywuzzy.

35.  Rebora PS, Agus; Reilly, Marie. bshazard. 1.1 ed2018. CRAN; 2018 [cited 2021 August 28]. Available from: https://cran.r-project.org/web/packages/bshazard/index.html.

**Reference 52: Please cite preprints per the guidelines here -https://journals.plos.org/plosmedicine/s/submission-guidelines#loc-references**

We have edited this citation as follows:

Walter KS, dos Santos PCP, Gonçalves TO, da Silva BO, da Silva Santos A, de Cássia Leite A, et al. Genomic evidence for prisons as amplifiers of community tuberculosis epidemics. medRxiv [Preprint]. 2021 [cited 2021 August 28]. doi: 10.1101/2021.06.22.21259360.

**Please ensure that the study is reported according to the STROBE guideline, and include the completed STROBE checklist as Supporting Information. Please add the following statement, or similar, to the Methods: "This study is reported as per the Strengthening the Reporting of Observational Studies in Epidemiology (STROBE) guideline (S1 Checklist)."**

**The STROBE guideline can be found here:** [**http://www.equator-network.org/reporting-guidelines/strobe/**](http://www.equator-network.org/reporting-guidelines/strobe/)

**When completing the checklist, please use section and paragraph numbers, rather than page numbers.**

We have added the STROBE checklist as S1 Checklist (pasted below). We have added the following statement to the methods (line 207): “We report this study as per the Strengthening the Reporting of Observational Studies in Epidemiology (STROBE) guidelines (S1 Checklist).”

|  | **Item No** | **Recommendation** | **Section (Paragraph #)** |
| --- | --- | --- | --- |
| **Title and abstract** | 1 | (*a*) Indicate the study’s design with a commonly used term in the title or the abstract | Title, abstract |
|  |  | (*b*) Provide in the abstract an informative and balanced summary of what was done and what was found | Abstract |
| **Introduction** | | | |
| Background/rationale | 2 | Explain the scientific background and rationale for the investigation being reported | Introduction (1-4) |
| Objectives | 3 | State specific objectives, including any prespecified hypotheses | Introduction (5) |
| **Methods** | | | |
| Study design | 4 | Present key elements of study design early in the paper | Methods (1-2) |
| Setting | 5 | Describe the setting, locations, and relevant dates, including periods of recruitment, exposure, follow-up, and data collection | Methods (1-2, 6) |
| Participants | 6 | (*a*) Give the eligibility criteria, and the sources and methods of selection of participants. Describe methods of follow-up | Methods (1-2, 5-6), Appendix (1-4) |
|  |  | (*b*) For matched studies, give matching criteria and number of exposed and unexposed | N/A |
| Variables | 7 | Clearly define all outcomes, exposures, predictors, potential confounders, and effect modifiers. Give diagnostic criteria, if applicable | Methods (2, 5-6) |
| Data sources/ measurement | 8* | For each variable of interest, give sources of data and details of methods of assessment (measurement). Describe comparability of assessment methods if there is more than one group | Methods (8-11), Appendix (5, 8, 9) |
| Bias | 9 | Describe any efforts to address potential sources of bias | Appendix (7-8, 12) |
| Study size | 10 | Explain how the study size was arrived at | Methods (1-2, 5) |
| Quantitative variables | 11 | Explain how quantitative variables were handled in the analyses. If applicable, describe which groupings were chosen and why | Methods (9, 12) |
| Statistical methods | 12 | (*a*) Describe all statistical methods, including those used to control for confounding | Methods (9, 12-14), Appendix (11) |
|  |  | (*b*) Describe any methods used to examine subgroups and interactions | Methods (12-14) |
|  |  | (*c*) Explain how missing data were addressed | Methods (8, 10, 12), Appendix (1-5, 8) |
|  |  | (*d*) If applicable, explain how loss to follow-up was addressed | Appendix (7) |
|  |  | (*e*) Describe any sensitivity analyses | Appendix (12) |
| **Results** | | |  |
| Participants | 13* | (a) Report numbers of individuals at each stage of study—eg numbers potentially eligible, examined for eligibility, confirmed eligible, included in the study, completing follow-up, and analysed | Results (1) |
|  |  | (b) Give reasons for non-participation at each stage | Appendix (8) |
|  |  | (c) Consider use of a flow diagram | Figs. 1, S2 |
| Descriptive data | 14* | (a) Give characteristics of study participants (eg demographic, clinical, social) and information on exposures and potential confounders | Table 1 |
|  |  | (b) Indicate number of participants with missing data for each variable of interest | Methods (7) |
|  |  | (c) Summarise follow-up time (eg, average and total amount) | Results (1) |
| Outcome data | 15* | Report numbers of outcome events or summary measures over time | Results (1), Table 1 |
| Main results | 16 | (*a*) Give unadjusted estimates and, if applicable, confounder-adjusted estimates and their precision (eg, 95% confidence interval). Make clear which confounders were adjusted for and why they were included | Results (3-6) |
|  |  | (*b*) Report category boundaries when continuous variables were categorized | Methods (12) |
|  |  | (*c*) If relevant, consider translating estimates of relative risk into absolute risk for a meaningful time period | Results (3-6) |
| Other analyses | 17 | Report other analyses done—eg analyses of subgroups and interactions, and sensitivity analyses | Results (3-8), Discussion: “Limitations” |
| **Discussion** | | | |
| Key results | 18 | Summarise key results with reference to study objectives | Discussion (1) |
| Limitations | 19 | Discuss limitations of the study, taking into account sources of potential bias or imprecision. Discuss both direction and magnitude of any potential bias | Discussion (9) |
| Interpretation | 20 | Give a cautious overall interpretation of results considering objectives, limitations, multiplicity of analyses, results from similar studies, and other relevant evidence | Discussion (2-7) |
| Generalisability | 21 | Discuss the generalisability (external validity) of the study results | Discussion (9) |
| **Other information** | | | |
| Funding | 22 | Give the source of funding and the role of the funders for the present study and, if applicable, for the original study on which the present article is based | Article meta-data |

**Comments from Reviewers:**

**Reviewer #1: Many thanks authors for their great effort to improve the manuscript. The authors have addressed my comments professionally. I am satisfied with the response and revision. Only a minor point, for table 1. all the categorical variables need to be summarised as count and percentage e.g. xx (yy%). Please just correct this and no need to come back to me. No other issues needing attention and I recommend publicaiton of the paper.**

Response #1: We thank the reviewer for their excellent feedback to improve our manuscript and for their suggestion about Table 1. We have edited Table 1 to add percentages of individuals with intervals in each location in the format that the reviewer describes. We did not have individual-level age data, but rather modeled the age distribution from aggregate data, so we are unable to show baseline age in terms of counts. One option is to show person-years in each age group as generated by our methods for person-time calculation and age structure estimation. We would prefer to only show percentages for the age distribution, as that is the format in which we retrieved the age structure from DEPEN; however, we are happy to include the breakdown of person-years by age group if the reviewer and editors recommend.

|  | | **Men and boys (N=105,465)** | **Women (N=9,286)** |
| --- | --- | --- | --- |
| **Number of individuals (%) with 1+ intervals in each location** | Closed prisons | 60,285 (57.2) | 7,715 (83.1) |
|  | Semi-open prisons | 26,667 (25.3) | 3,233 (34.8) |
|  | Police stations | 72,339 (68.6) | 4,332 (46.7) |
|  | Youth detention | 4,939 (4.7) | 18 (0.2) |
|  | Post-release | 60,184 (57.1) | 8,347 (89.9) |
| **Median duration in days (IQR)** | Closed prisons | 153 (21-473) | 184 (34-482) |
|  | Semi-open prisons | 62 (13-169) | 93 (29-240) |
|  | Police stations | 3 (1-17) | 2 (1-21) |
|  | Youth detention | 229 (27-758) | 300 (46-498) |
|  | Post-release | 348 (50-1300) | 849 (131-2262) |
| **Percentage of incarcerated population in each age group** | 18-24 | 23.3 | 26.5 |
|  | 25-29 | 24.4 | 20.6 |
|  | 30-34 | 19.8 | 19.0 |
|  | 35-45 | 22.3 | 23.0 |
|  | 46-60 | 8.9 | 9.8 |
|  | 61+ | 1.3 | 1.1 |
| **Number of deaths (% of total deaths)** | Under 18 | 27 (0.9) | 0 (0) |
|  | 18-24 | 423 (14.5) | 14 (6.4) |
|  | 25-29 | 396 (13.6) | 22 (10.0) |
|  | 30-34 | 389 (13.4) | 23 (10.5) |
|  | 35-45 | 658 (22.6) | 73 (33.2) |
|  | 46-60 | 608 (20.9) | 54 (24.6) |
|  | 61+ | 374 (12.9) | 31 (14.1) |
|  | Unreported | 33 (1.1) | 2 (0.9) |
|  | Total | 2908 | 219 |

**Reviewer #2: I appreciate the authors' attention to my comments, especially my concerns about causality. I am satisfied with the revisions they have carried out.**

Response #2: We thank the reviewer for their excellent suggestions to improve our manuscript.
